# Supplementary material for: Miniaturization of CRISPR/Cas12-Based DNA Sensor Array by Non-Contact Printing
Source: Micromachines (Basel). 2024 Jan 17;15(1):144. doi: 10.3390/mi15010144 (PMC10818962; doi:10.3390/mi15010144)
Supplement: Supplementary file 1 [file micromachines-15-00144-s001.zip › micromachines-2807065-supplementary.pdf]

Supplementary Materials

Miniaturization of CRISPR/Cas12-Based DNA Sensor Array by Non-Contact Printing

Hiroki Shigemori [1,2], Satoshi Fujita [1], Eiichi Tamiya [1,3] and Hidenori Nagai [1,2,*]

[1] Advanced Photonics and Biosensing Open Innovation Laboratory (PhotoBIO-OIL), National Institute of Advanced Industrial Science and Technology (AIST), Photonics Center Osaka University, 2-1 Yamada-Oka, Suita 565-0871, Osaka, Japan; h.shigemori@aist.go.jp (H.S.); s-fujita@aist.go.jp (S.F.); tamiya.handai-oil@aist.go.jp (E.T.)

[2] Graduate School of Human Development and Environment, Kobe University, 3-11 Tsurukabuto, Nada-ku, Kobe 657-0011, Hyogo, Japan

[3] Institute of Scientific and Industrial Research (SANKEN), Osaka University, 8-1 Mihogaoka, Ibaraki 567-0047, Osaka, Japan

* Correspondence: hide.nagai@aist.go.jp; Tel.: +81-72-751-9527

Figure S1: Process to wash the bottom surface of the 96-well ···· S2
Figure S2: Extraction workflow of the Cas12-immobilized region from the FAM image ···· S2
Figure S3: HEX-fluorescence response to target dsDNA concentration in each printing volume condition ···· S3
Figure S4: One-pot triple-target dsDNA detection on the non-contact-patterned SPCC-based sensor array fabricated by the optimized process ···· S4
Figure S5: Proximal spot-dispensing model of inkjet patterning and the calculation method of its spot-density ···· S4

Table S1: Oligonucleotide sequences of amplified target dsDNA, primer, synthesized target dsDNA, crRNA, FAM-crRNA, and ssDNA reporter ···· S5
Table S2: Components of PCR mixture ···· S6
Table S3: Conditions of non-contact printing of the Cas12-crRNA droplet ···· S6

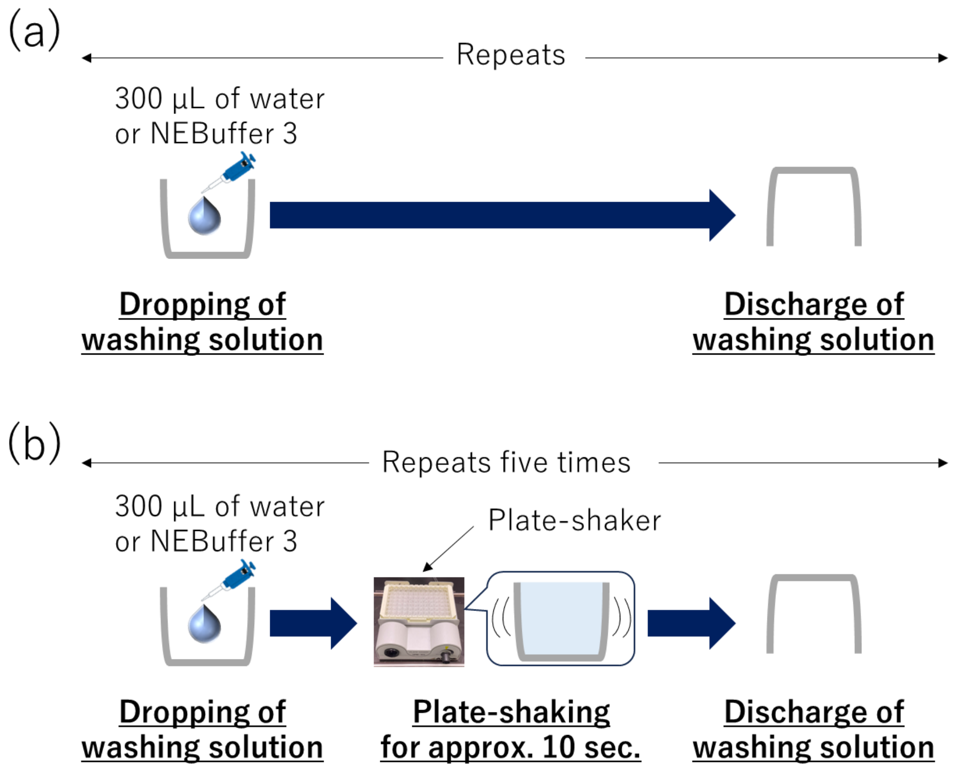

Figure S1. Process to wash the bottom surface of the 96-well: (a) 3.3 section and later, (b) Up to 3.2 section

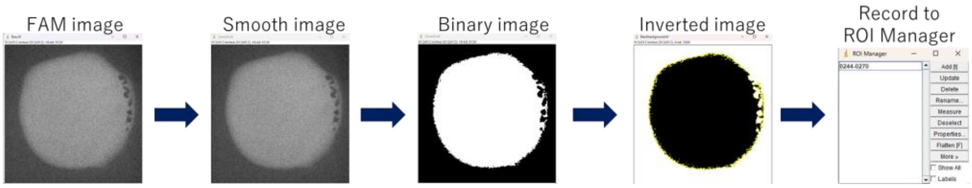

Figure S2. Extraction workflow of the Cas12-immobilized region from the FAM image

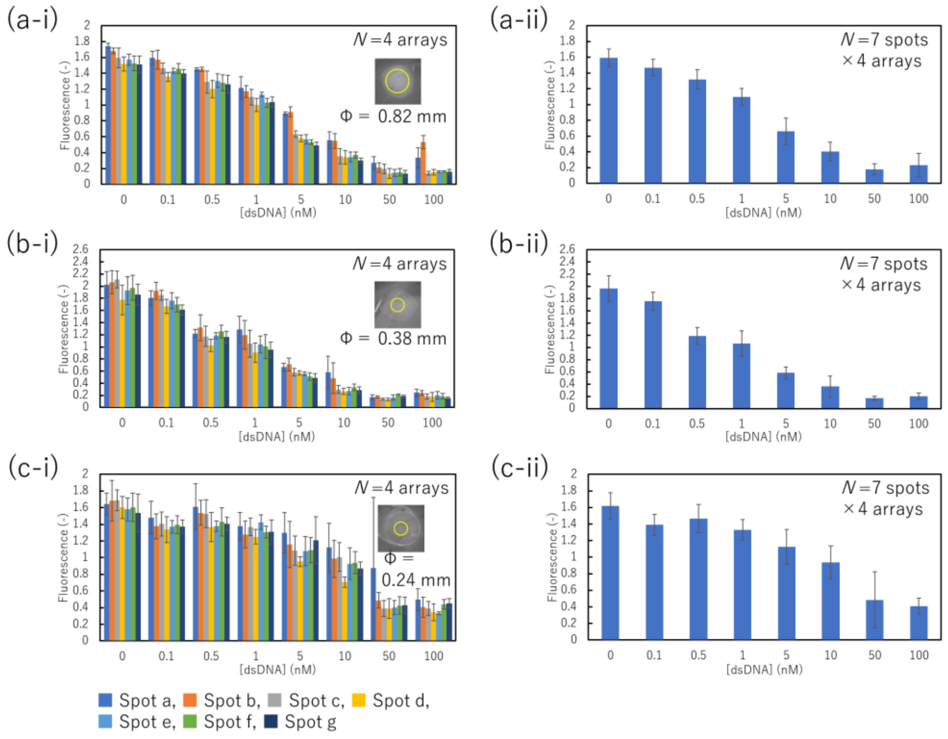

Figure S3. HEX-fluorescence response to target dsDNA concentration in each printing volume condition: (a-i, b-i and c-i) HEX-fluorescence intensity at the center part of each spot (the yellow-circled position) in 40, 20 and 10 nL printing volume condition, respectively, (a-ii, b-ii and c-ii) HEX-fluorescence intensity of each [dsDNA] in 40, 20 and 10 nL printing volume condition, respectively

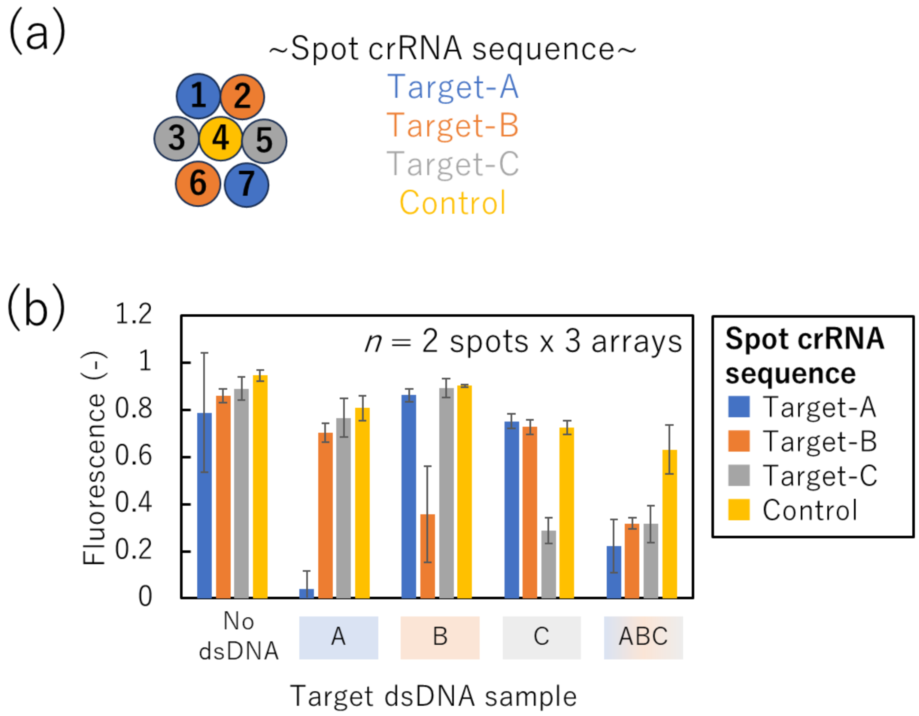

Figure S4. One-pot triple-target dsDNA detection on the non-contact-patterned SPCC-based sensor array fabricated by the optimized process: (a) crRNA sequence of each spot, (b) HEX-fluorescence intensity in each crRNA sequence condition after the incubation of each dsDNA sample (fluorescence intensity was extracted at the region with a radius of 0.71 mm around the center of each spot)

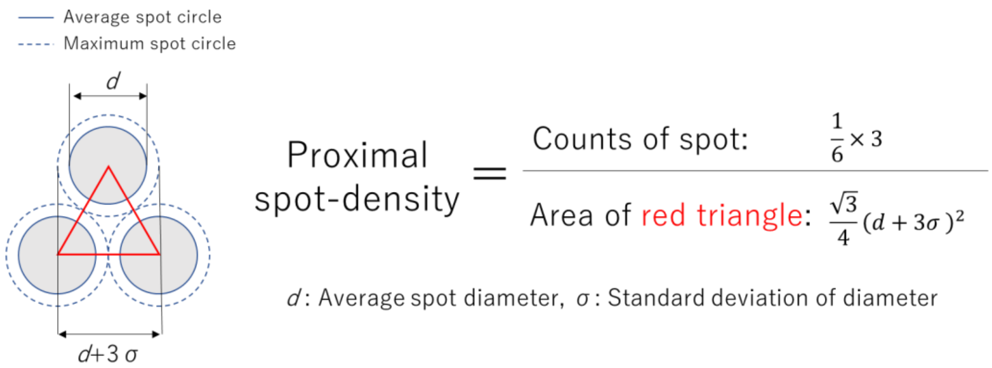

Figure S5. Proximal spot-dispensing model of inkjet patterning and the calculation method of its spot-density

Table S1. Oligonucleotide sequences of amplified target dsDNA, primer, synthesized target dsDNA, crRNA, FAM-crRNA, and ssDNA reporter (the [ACC] sequence of target dsDNA amplicon was converted to [TTT] (part of the PAM sequence) by forward primer; Underline indicates the targeted region of the dsDNA and the target-recognizing region of the crRNA; FAM, HEX, AmC7, and BHQ1 stand for 6-carboxyfluorescein, Hexachloro-fluorescein, Amino Modifier C7 and Black Hole Quencher 1).

**Amplified region of target dsDNA (sense strand)**	**Sequence (5'→3')**
pEGFP-N1	CACCTACGGCAAGCTG[ACC]CTGAAGTTCATCTGCACCACCGGCAA-GCTGCCCGTGCCCTGGCCCACCCTCGTGAC-CACCCTGACCTACGGCGTGCAGTGCTTCAGCCGCTACCCCGACCACATGAAGCAGCACGACTTCTTCAAGTCCGCCATGCCCGAAGGCTACGTCCAGGAGCGCACCATCTTCTTCAAGGACG
pVenus-N1	CACCTACGGCAAGCTG[ACC]CTGAAGCTGATCTGCACCACCGGCAA-GCTGCCCGTGCCCTGGCCCACCCTCGTGAC-CACCCTGGGCTACGGCCTGCAGTGCTTCGCCCGCTACCCCGACCACATGAAGCAGCACGACTTCTTCAAGTCCGCCATGCCCGAAGGCTACGTCCAGGAGCGCACCATCTTCTTCAAGGACG
**Primer**	
Forward	CACCTACGGCAAGCTG[TTT]CTGA
Reverse	CGTCCTTGAAGAAGATGGTGCGC
**Synthesized target dsDNA**	
Target-A	AATTGCACAATTTGCTCCAAGTGCCTCTGCATTCTTTGGAATGT
Target-B	AGGAGTGTCTTTTCAATTACTTGGGTGTGACCCTGAAGACTCGGA
Target-C	AATTGCACAATTTGCCCCCAGCGCTTCAGCGTTCTTCGGAATGTCGC
**crRNA**	
For pVenus-N1	UAAUUUCUACUAAGUGUAGAUUGAAGCUGAUCUGCACC
**FAM-crRNA**	
For pVenus-N1	UAAUUUCUACUAAGUGUAGAUUGAAGCUGAUCUGCACC -FAM
Negative control	UAAUUUCUACUAAGUGUAGAUUGAAGUAGAUAUGGCAGCAC -FAM
For target-A	UAAUUUCUACUAAGUGUAGAUCUCCAAGUGCCUCUGCAUUC -FAM
For target-B	UAAUUUCUACUAAGUGUAGAUAAUUACUUGGGUGUGACCCU -FAM
For target-C	UAAUUUCUACUAAGUGUAGAUCCCCCAGCGCUUCAGCGUUC -FAM
**ssDNA reporter**	
HEX-Poly T-NH2-20nt	HEX-TTTTTTTTTTTTTTTTTTTT-AmC7
HEX-Poly T-NH2-40nt	HEX-TTTTTTTTTTTTTTTTTTTTTTTTTTTTTTTTTTTTTTTT -AmC7
HEX-Poly T-NH2-60nt	HEX-TTTTTTTTTTTTTTTTTTTTTTTTTTTTTTTTTTTTTTTTTTTTTTTTTTTTTTTTTTTT -AmC7
HEX-Poly T-NH2-80nt	HEX-TTTTTTTTTTTTTTTTTTTTTTTTTTTTTTTTTTTTTTTTTTTTTTTTTTTTTTTTTTTTTTTTTTTTTTTTTTTTTTTT -AmC7

HEX-Poly T-NH2-100nt	HEX-TTTTTTTTTTTTTTTTTTTTTTTTTTTTTTTTTTTTTTTTTTTTTTTTTTTTTTTTTTTTTTTTTTTTTTTTTTTTTTTTTTTTTTTTTTTTTTTTTTTTT-AmC7
F-Q ssDNA reporter	HEX-TTATT-BHQ1

Table S2. Components of PCR mixture. The reagents were added to tubes in order from top to bottom of the list. The 10x Fast Buffer I and dNTP mixture were part of the SpeedSTAR HS Polymerase kit

**Reagent**	**Concentration**
**Nuclease-free water**	—
**10x Fast buffer I**	1x
**dNTP mixture (2.5 mM each)**	200 μM
**Forward primer**	0.4 μM
**Reverse primer**	0.4 μM
**SpeedSTAR HS Polymerase**	25 mU μL^{-1}
**pVenus-N1 plasmid**	50 fg μL^{-1}

Table S3. Conditions of non-contact printing of the Cas12-crRNA droplet

**Conditions**		**Before 3.4. section**	**3.4. and 3.5. section**
Syringe pressuring speed	Initiate (μL/s)	1	0.2
	Maximum (μL/s)	10	1
	Accelaration (μL/s2)	200	100
Pre-pressure before the opening of the solenoid valve (μL)		0.65	
Opening time of the solenoid valve (μs)		650	
Printing volume (nL)		50	10 or 20 or 40

Table S4. Correspondence table of positive (>LOD) / negative (<LOD) samples and their test results, and calculation results of sensitivity (positive results ratio from positive samples) and specificity (negative results ratio from negative samples).

Table S4a. Results in 40 nL-printing volume condition (0, 0.1, 0.5 nM dsDNA samples are defined as negative; 1, 5, 10, 50, 100 nM dsDNA samples are defined as positive)

	**Positive samples (>LOD)**	**Negative Samples (<LOD)**
**Positive results (>3σ)**	20	1
**Negative results (<3σ)**	0	11
	**Sensitivity: 100%**	**Specificity: 92%**

Table S4b. Results in 20 nL-printing volume condition (0, 0.1, 0.5 nM dsDNA samples are defined as negative; 1, 5, 10, 50, 100 nM dsDNA samples are defined as positive)

	**Positive samples (>LOD)**	**Negative Samples (<LOD)**
**Positive results (>3σ)**	20	4
**Negative results (<3σ)**	0	8
	**Sensitivity: 100%**	**Specificity: 67%**

Table S4c. Results in 10 nL-printing volume condition (0, 0.1, 0.5, 1 nM dsDNA samples are defined as negative; 5, 10, 50, 100 nM dsDNA samples are defined as positive)

	**Positive samples (>LOD)**	**Negative Samples (<LOD)**
**Positive results (>3σ)**	16	0
**Negative results (<3σ)**	0	16
	**Sensitivity: 100%**	**Specificity: 100%**
